# Prodromal Markers of Parkinson's Disease in Patients With Essential Tremor

**DOI:** 10.3389/fneur.2020.00874

**Published:** 2020-08-25

**Authors:** Xi-Xi Wang, Ya Feng, Xuan Li, Xiao-Ying Zhu, Daniel Truong, William G. Ondo, Yun-Cheng Wu

**Affiliations:** ^1^Department of Neurology, Shanghai General Hospital, Shanghai Jiao Tong University School of Medicine, Shanghai, China; ^2^Shanghai General Hospital of Nanjing Medical University, Nanjing, China; ^3^Orange Coast Memorial Medical Center, The Truong Neurosciences Institute, Fountain Valley, CA, United States; ^4^Department of Neurosciences and Psychiatry, University of California, Riverside, Riverside, CA, United States; ^5^Weill Cornell Medical School, Methodist Neurological Institute, Houston, TX, United States

**Keywords:** essential tremor, Parkinson's disease, ET-PD, prodromal markers, substantia nigra hyperechogenicity

## Abstract

**Background:** Essential tremor (ET) is manifested as an isolated syndrome of bilateral upper limb action tremor. Parkinson's disease (PD) is the second most common neurodegenerative disease, with typical motor symptoms of bradykinesia, rigidity, and resting tremor. ET-PD describes the new-onset of PD in ET patients. Recently, numerous studies on epidemiology, genetics, pathology, clinical features, and neuroimaging studies are challenging the idea that ET is an isolated disease, suggesting that patients with ET have the tendency to develop PD.

**Methods:** In this review article, we collected recent findings that reveal prodromal markers of PD in patients with ET.

**Results:** Substantia nigra hyperechogenicity serves as a prodromal marker for predicting the development of PD in patients with ET and provides a reference for therapeutic strategies. Additional potential markers include other neuroimaging, clinical features, heart rate, and genetics, whereas others lack sufficient evidence.

**Conclusion:** In consideration of the limited research of PD in patients with ET, we are still far from revealing the prodromal markers. However, from the existing follow-up studies on ET patients, Substantia nigra hyperechogenicity may enable further exploration of the relationship between ET and PD and the search for pathogenesis-based therapies.

## Introduction

Essential tremor (ET) is defined as an isolated postural and/or kinetic tremor syndrome of the bilateral upper limbs, with or without tremor in other locations (e.g., head, voice, or lower limbs). The traditional idea proposes that no other neurological signs such as dystonia, ataxia, or parkinsonism are present (1–3), although ET is sometimes considered a diagnostic of exclusion (4). With accumulated studies on ET, researchers today recognize the concept of ET plus (3), which refers to tremor with the characteristics of ET and additional neurological signs such as impaired tandem gait, questionable dystonic posturing, memory impairment, or other mild neurologic signs. However, these additional neurological signs are of unknown significance and are insufficient to make an additional syndrome classification or diagnosis. Dividing ET into two categories (ET and ET plus) is helpful in determining pathophysiological characteristics and therapeutic strategies.

Parkinson's disease (PD) is the second most common neurodegenerative disease, characterized by the degeneration of dopaminergic neurons in the substantia nigra (SN) and reduced dopamine levels in the midbrain (5–8). The pathological hallmark of PD is the presence of Lewy bodies, and the classical motor symptoms of PD are bradykinesia, rigidity and resting tremor, and numerous non-motor symptoms including constipation, hyposmia/anosmia, rapid eye movement sleep behavior disorder (RBD), and so on (9).

ET-PD referred to in this article means new onset of PD in patients previously diagnosed with ET. Essential tremor has been traditionally viewed as a “benign” disease; however, accumulated data from the studies of clinical characteristics, epidemiology, neuroimaging, genetics, and pathology support a poorer prognosis than originally believed (10–12). Evidence indicates that patients with ET are four times more likely to develop PD than those without baseline ET (13). Additionally, nigrostriatal degeneration developed before the onset of motor symptoms of parkinsonism (14, 15). Sensitive and effective prodromal markers predicting which ET patients will subsequently develop PD are needed to further understand the biological nature of ET, which will provide a pathology-based categorization, diagnosis, prognosis, and eventual treatment of ET.

## Neuroimaging

### Transcranial Sonography

Transcranial sonography (TCS) can show the structure of the brain parenchyma and reveal the lesions of the SN. It is anecdotally viewed as a non-invasive tool that can be utilized to detect the intensity of the SN and measure the ratio of the hyperechoic area to midbrain area (S/M ratio). The SN hyperechogenicity refers to the high intensity and large area (>0.8–0.2 cm^2^) of the echo in the SN shown by transcranial ultrasound.

As early as 1995 (16), scientists first described the mysterious relationship between SN hyperechogenicity and PD, which provided a new perspective and direction for research. It was verified that SN hyperechogenicity as reflected by TCS, seemed to be a prodromal marker for PD (17–20). In 2007, research including 164 healthy Taiwanese, 40 early-onset PD patients, and 40 late-onset PD patients focused on the area of SN hyperechogenicity and the S/M ratio (21). The results indicated that the S/M ratio is a more sensitive marker than SN hyperechogenicity in diagnosing PD. Based on the investigation that tremor-dominant PD (TDPD) patients had significantly higher nigral R2^*^ relaxation rate values in magnetic resonance imaging (MRI) (34.1 ± 5.7) than those with tremor in dystonia (30.0 ± 3.9), ET (30.6 ± 4.8), and controls (30.0 ± 2.8). This and other research confirm that increased iron content in the SN is significantly associated with PD. Afterward, combining iron metabolism and the features of TCS (22, 23), researchers presumed that the change of the echogenicity in the SN may be attributed to oxidative stress and the injury of neurons caused by the fluctuation of iron content. In a prospective multicenter study, researchers also demonstrated that the elderly with SN hyperechogenicity had a higher risk of developing PD (24).

Transcranial sonography measurement of the midbrain is a sensitive and specific preliminary screening method to identify certain population with higher risk of developing PD. A small trial also supported its use at predicting ET patients who will develop PD, but this needs to be replicated in larger trials (25). After performing TCS of the SN and clinical examination in 80 PD patients, 30 ET patients, and 80 age- and gender-matched controls, researchers realized that SN hyperechogenicity may be associated with an increased risk developing PD later in life or might be due to the damage of areas near the nucleus ruber in ET patients (26). Subsequently, Sprenger et al. (27) conducted a prospective cohort study in 70 ET patients, which evaluated demographics, TCS, Bain tremor scale, and Movement Disorder Society-Sponsored Revision of the Unified Parkinson's Disease Rating Scale. After an average of 6.16 years' follow-up, they identified nine ET-PD patients, seven of whom had SN hyperechogenicity greater than baseline. Statistical analysis showed that the relative risk of developing PD in patients with ET who had hyperechogenicity at baseline vs. those without hyperechogenicity was 7.00 (27). Moreover, a recent longitudinal study demonstrated that after 3 years' follow-up, 9 of 59 ET patients developed clinical features meeting diagnostic criteria for probable PD (ET-PD), and this group had a significantly greater SN hyperechogenicity at baseline from healthy controls (28).

Several studies have consistently demonstrated that SN hyperechogenicity on TCS is a prodromal marker for development of PD in ET patients (29, 30). It serves as a visualization tool for the diagnosis and prognosis of ET patients. Different characteristics of the echo are associated with not only the diagnosis of PD but also the therapeutic response (31). Hence, in addition to differential diagnosis between PD and other movement disorders (32), SN hyperechogenicity can also serve as a prodromal marker for predicting the development of PD in patients with ET and provide a guidance for therapeutic strategy (Table 1).

**Table 1:** Studies on SN hyperechogenicity indicate a role in prediction of the risk of PD.

**Time & Country & Reference**	**Subjects**	**Results**
1995. Germany (16)	30 patients with PD and 30 age- and sex-matched non-Parkinsonian controls	The degree of hyperechogenicity of the SN closely correlated with the severity and duration of PD.
2001. Germany (19)	93 subjects older than 60 years without history of extrapyramidal disorder	With increasing age, subjects with SN hyperechogenicity develop a more substantial slowing of movements than subjects without this echo pattern, stressing the functional relevance of this sonographic finding.
2004. Germany (18)	7 symptomatic and 7 asymptomatic PMC from large kindred with adult-onset parkinsonism	SN hyperechogenicity is an early marker to detect preclinical Parkinsonism.
2007. Taiwan (21)	164 healthy Taiwanese, 40 EOPD patients, and 40 LOPD patients	S/M ratio is a more sensitive measure than SN hyperechogenicity in diagnosing PD.
2011. Germany (20)	1,847 participants who are 50 years or older without evidence of PD or any other neurodegenerative disease	A highly increased risk for PD in elderly individuals with SN hyperechogenicity.
2013. Germany (24)	1,271 of the initial 1,847 at baseline PD-free participants 50 years or older,	SN hyperechogenicity is an important risk marker for PD.
2007. Austria (25)	44 ET patients with 100 controls and 100 PD patients	An increased risk of ET patients to develop PD.
2016. Austria & Germany (27)	70 patients suffering from ET	SN hyperechogenicity is also associated with an increased risk for PD in patients with ET.
2019. Italy (28)	79 with PD, 59 with ET and 50 matched controls	SN hyperechogenicity in ET seems to represent a risk marker for developing early parkinsonian symptoms or signs in the 3 years following TCS assessment.

*SN, substantia nigra; PD, Parkinson's disease; PMC, parkin mutation carriers; EOPD, early-onset PD; LOPD, late-onset PD; S/M ratio, the ratio of hyperechoic area to midbrain area; ET, essential tremor*.

### Other Neuroimaging

Brain structural and functional neuroimaging methods might show abnormalities in ET patients. Voxel-based morphometry (VBM) is commonly used to learn about gray matter and white matter size. Benito-Leon et al. (33) found significant white matter changes in right cerebellum, left medulla, right parietal lobe, and right limbic lobe, as well as gray matter alteration in bilateral cerebellum, bilateral parietal lobe, right frontal lobe, and right insula in ET patients compared with 20 age- and gender-matched healthy controls. Lin et al. (34) compared VBM in 10 ET patients with 10 PD patients and revealed that PD and ET caused specific patterns of brain volume alterations in the examined brains. The brain volume of the ET group was significantly smaller in the thalamus and the middle temporal gyrus and larger of the gray matter in the middle frontal gyrus, the middle temporal gyrus, and the cerebellum posterior lobe than that of the PD group (34). These studies suggested comprehensive changes in the gray and white matter of brain in ET patients.

Diffusion tensor imaging (DTI) derived mean diffusivity (MD) and fractional anisotropy. It exhibits great fidelity in showing brain microstructure and connections. A DTI-based study performed in 67 ET patients (29 ET with and 38 without resting tremor) and 39 age-matched healthy controls indicated that MD was significantly higher in the cerebellar gray matter in the ET group. It demonstrates that ET patients have cerebellum microstructural changes, and some other networks alterations may exist in the development of ET (35).

After enrolling 15 ET patients with resting tremor and 15 TDPD patients, Cherubini et al. (36) did a combination of VBM and DTI to distinguish these two groups with a ground truth of Dopamine transporter 123I-FP-CIT-single-photon emission tomography (DAT-SPECT), and they found the combination shows 100% accuracy in differentiating these two groups.

Proton MR spectroscopy (^1^H-MRS) is a non-invasive, quantitative technique that reflects the neurometabolic alterations *in vivo*. There are many biochemical markers including NAA/Cr (a neural density marker), Glx/Cr (an intracellular neurotransmitter marker), and Cho/Cr (a membrane marker). In 2002, researchers measured 16 ET patients and 11 controls with MRS and found that cortical NAA/Cr in the cerebellar was reduced in ET cases, and the value was inversely proportional to arm tremor severity (37). With the application of a 3-T scanner, Barbagallo et al. (38) found a statistically significant increase in Glx and Glx/Cr values in 16 ET patients in both thalami compared to 14 healthy controls, and the tremor severity was directly proportional to these two values. These results suggested that increased thalamic glutamatergic transmission and cerebellum play a role in the pathogenesis in ET. In addition to these imaging techniques (Table 2), MRI imaging focused on brain iron deposition, and functional MRI and other neuroimaging methods also indicated the details of the involved brain networks in ET and provided some help to differentiate mixed tremor, ET, and PD (39–41). Even though some of the research did not explore prodromal markers of PD in ET patients from a longitudinal-study perspective, it still reflects the relationship between the two disorders, provides methods in differential diagnosis, and promotes the knowledge of behind pathophysiologic mechanisms.

**Table 2:** Studies on other neuroimaging about prodromal markers of new-onset PD in ET patients.

	**Neuroimaging techniques**	**Subjects**	**Results**	**References**
Structural neuroimaging	VBM	ET vs. healthy controls	White and gray matter of many brain regions changed in ET patients	(33)
		ET vs. PD	PD and ET caused specific patterns of brain volume alterations	(34)
	DTI	ET vs. healthy controls	MD was significantly higher in the cerebellar gray matter of ET group, indicating cerebellum microstructural changes and some other networks involved	(35)
	VBM+DTI	ET vs. PD	Combination of two methods helps in differentiating two kinds of patients	(36)
Metabolic neuroimaging	1H-MRS	ET vs. healthy controls	Cortical NAA/Cr in the cerebellar was reduced in ET cases and the value was inversely proportional to arm tremor severity	(37)
		ET vs. healthy controls	Glx and Glx/Cr values increased in both thalami of ET patients and the value was proportional to tremor severity, indicating thalamic glutamatergic transmission and cerebellum invloved	(38)
	Iron deposition	ET vs. healthy controls	Iron accumulation increased in ET patients, suggesting a possible involvement of motor systems outside of the cerebellar pathway	(39)
Functional neuroimaging	DAT-SPECT+MIBG	Mixed tremor vs. PD vs. ET vs. controls	Combined use of these two techniques can help distinguish ET patients from PD patients and parkinsonism	(40)
	fMRI	ET VS healthy controls	Cerebellar neurodegeneration underlying essential tremor is reflected in abnormal communications between key working memory regions and that adaptive mechanisms involved in to influence cognition	(41)

*VBM, voxel-based morphometry; PD, Parkinson's disease; ET, essential tremor; DTI, Diffusion tensor imaging; MD, Mean diffusivity; 1H-MRS, Proton MR Spectroscopy; NAA, N-acetyl-aspartate + N-acetyl-aspartyl-glutamate; Cr, creatine + phosphocreatine; Glx, glutamate + glutamine; DAT-SPECT, Dopamine transporter 123I-FP-CIT-single-photon emission tomography; MIBG, myocardial scintigraphy with 123metaiodobenzylguanidine*.

## Clinical Characteristics

Identifying clinical characteristics that increase the risk of developing PD in ET patients may help in the development of potential disease-modifying therapies. Essential tremor classically presents as a bilateral but asymmetric kinetic and postural tremor of the upper limbs, head, voice, or a combination. Parkinson disease is generally manifested as asymmetric symptoms of bradykinesia, rigidity, resting tremor, and later postural instability. Many studies have summarized the similarities and differences of the two diseases based on clinical characteristics (i.e., motor and non-motor symptoms) (30, 42, 43), but there are still few prodromal markers with respect to the clinical characteristics of ET-PD patients, and many of them are still in the conjecture stage (Table 3).

**Table 3:** Clinical characteristics which can predict the risk of ET patients developing into PD.

**Clinical characteristics**	**Prodromal markers**	**References**
Tremor	Circumscribed resting tremor	(44)
	Late onset asymmetrical postural tremor	(45)
	Jaw tremor in ET	(46, 47)
	A kinematic data: TSI	(48)
Non-motor symptoms	Olfactory decline (hyposmia/anosmia)	(49–51)
	Cognitive decline	(52–54)
	Sleep disorder, especially RBD	(55–59)
	RBD & autonomic symptoms	(60)

*ET, essential tremor; PD, Parkinson's diseases; TSI, the tremor stability index; RBD, REM sleep behavior disorder*.

### Tremor

An epidemiological survey showed that tremor of the upper limbs was presented in more than 95% of the ET patients, followed by the head (>30%), voice (>20%), tongue (20%), face and/or jaw (10%), lower limbs (10%) and trunk (5%) (61, 62). Essential tremor and PD tremor are mainly identified based on aspects of location, frequency, and form. However, few studies have identified risk markers based on tremor characteristics in ET patients who have the potential to develop PD.

The location of tremor in the two groups was most common in upper limbs, whereas the prevalence of resting tremor in ET-PD patients was higher than that in patients with isolated ET, and the tremor distribution was more limited in ET-PD (*p* < 0.05) (30). Another study involving 53 ET-PD patients and 150 ET patients noted a biased distribution toward male (67.9% male) of ET-PD, which is identical to that of PD (67.9% male) (44). The latency from the onset of ET to develop into PD ranged from short duration (<5 years, in 38.5%) to long duration (>20 years), with a mean duration of 14 ± 15 years. In ET-PD patients, the side of dominant initial ET severity usually coincides with that of dominant PD severity (*p* < 0.05). The initial cardinal sign was resting tremor in the vast majority of PD patients, which indicated that resting tremor may be an omen of PD (44). This evidence implied that circumscribed resting tremor may be a prodromal marker for ET patients to develop PD. However, some studies also pointed out that resting tremor (in the arms but not the legs) can occur as a late feature in ET patients (63), so it is worth considering the role of circumscribed resting tremor in the course of predicting PD in ET patients. Additionally, data from a retrospective observation of 13 patients initially diagnosed as ET based on tremor characteristics, alcohol responsiveness, and family history who met the PD criteria after a variable long latent period suggest that late-onset asymmetrical postural tremor may be a signal for developing PD in the long term even if there is no resting tremor (45).

In addition to upper limbs, tremors of the head, voice, and jaw also exist in ET with long disease duration (46). The incidence of jaw tremor ranges from 7.5 to 18% (47, 64, 65) in ET patients. Essential tremor patients with jaw tremor have more severe clinical symptoms and more widely distributed tremor than patients without it. Essential tremor patients with head tremor may be also regarded as a more severe clinical subtype (66). This manifestation combined with the hypothesis that the evolution from ET to PD is caused by the spread of Lewy bodies in the cerebellothalamocortical circuit leads us to speculate that there may be some relationship between jaw tremor and subsequent PD. Additional longitudinal studies are needed to assess whether jaw tremor in ET is a prodromal marker for subsequent PD.

In addition to the research on prodromal markers based on the position, range, and form of the tremor (67–70), a recent study investigated the motor feature: the Tremor Stability Index (TSI) (48). The TSI can be obtained by kinematics measurement of tremor activity and applied to the analysis of tremor characteristics. After testing a cohort comprising 16 rest tremor recordings in TDPD and 20 postural tremor recordings in ET, researchers found a difference in TSI between these two groups [mean 0.7 ± 0.175 (SEM) in TDPD, 1.9 ± 0.134 in ET, *t*_(34)_ = −5.481, *p* < 0.001]. This suggests electrophysiological methods may be helpful to evaluate the nature of the tremor and to explore the underlying central oscillator circuits from peripheral tremor. We can also examine additional parameters to evaluate the prognosis of ET patients.

### Non-motor Symptoms

Non-motor symptoms are increasingly recognized as an important part of ET. Essential tremor patients show a variety of non-motor symptoms, such as cognitive decline, mood disorder, and hearing loss (71–78). However, studies on non-motor symptoms as possible prodromal factors for developing PD in ET patients are mostly lacking.

There has been increasing interest in olfactory dysfunction because it was identified as a prodromal feature of PD. Just as in PD patients, olfactory decline (hyposmia or anosmia) becomes more evident as the disease progresses in ET patients (49, 79); however, there are also studies that show no olfactory loss in ET. In 2008, Louis et al. (50) found that higher blood harmane concentration was correlated with olfactory decline in 83 ET cases (62.5%, *p* < 0.001). As a cerebellar toxin, harmane reflects the relationship between olfactory decline and a part of pathophysiology in the cerebellum. In 2016, a large European multicenter study using the 16-item Sniffin' Sticks test (SS-16) found that olfactory performance was lower in PD patients compared with atypical parkinsonism and non-PD patients in all cohorts (each *p* < 0.001), and a set of eight smell reduction olfactory tests can be used as a rapid detection tool for PD (51). This calls for olfactory testing of ET patients and longitudinal studies to explore whether hyposmia can be used as a prodromal marker for the development of PD in ET patients.

It is still controversial whether cognition declines in ET. As early as 2001, Gasparini et al. (52) carried out a preliminary study focusing on performance on frontal lobe tasks of 27 ET patients, 15 PD patients, and 15 healthy controls, and they found significant impairments both in attention and conceptual thinking tasks in ET patients, but with no statistical difference between ET patients and PD patients. Consistent with this research, many subsequent studies identified cognitive impairments in ET patients (80, 81). In 2019, researchers enrolled 23 ET patients and 23 healthy controls to evaluate topological properties of brain function network with the aid of resting-state functional MRI, which revealed that changes took place in many regions including hippocampus in ET patients (82). By employing a structured neuropsychological battery to access cognition in 40 ET patients and 40 healthy controls, Prasad et al. (83) revealed that ET patients with cognitive impairment have significant volumetric abnormalities of specific brain regions including several hippocampal subfields.

Recently, by evaluating the hippocampal subregions of PD with cognitive decline and PD without cognitive decline, Xu et al. (84) found that the hippocampal CA2/3, CA4, and DG subfields appeared sensitive in groups of PD with cognitive decline longitudinally, and the volume loss of CA2/3 and CA4-DG correlated with the degree of cognitive impairment. With the assistance of quantitative susceptibility mapping, Thomas et al. (85) tracked cognitive changes in PD and identified lower Montreal Cognitive Assessment (MoCA) scores in the hippocampus and thalamus. Compared with PD patients, ET-PD patients have poorer cognitive performance. After enrolling 30 ET-PD patients and 53 age-matched PD patients, researchers found that both the cursory total score of Mini-Mental State Examination (*p* = 0.001) and the Telephone Interview for Cognitive Status (*p* < 0.001) were lower in ET-PD than in PD, as well as the subscores related to orientation (*p* < 0.001), language (*p* < 0.001), and working memory (*p* = 0.001) (53). It has also been demonstrated that hippocampal microstructural damage is related to subclinical memory impairment in ET patients (57). Considering that cognitive disorders with involvement of the hippocampus occurred both in ET and PD patients, and ET-PD patients have poorer cognitive performance than PD patients, the role of cognitive impairment in predicting the development of PD in ET patients is worth exploring in future research.

Numerous studies have indicated that sleep disturbance, especially RBD, may be an early prodromal marker for developing PD in ET patients (58–60, 86). One study using the RBD Screening Questionnaire (RBDSQ) revealed that ~0.5% in the general population suffered from RBD (43.5%) (87). Another study in which 92 ET patients were assessed on the Scales for Outcomes in Parkinson's Disease–Autonomic questionnaire to evaluate autonomic symptoms and the RBDSQ to assess the RBD symptoms with ≥5 as a cut-off value for probable RBD (pRBD), 26.4% of the ET patients had pRBD and 98.1% of them reported at least one autonomic symptom (88). This association suggested that a subgroup of ET patients with pRBD may be at higher risk of PD progression. Salsone et al. (89) proposed that the presence of RBD in ET could identify a specific clinical phenotype and demonstrated significantly reduced scores on memory of ET patients with RBD compared to those without RBD, indicating RBD in ET patients is associated with cognitive impairment. Because RBD is a risk marker of PD in healthy people, its role in prediction of PD in ET patients deserves further longitudinal study.

## Heart Rate Variability

Recent autonomic symptom questionnaire-based analyses and sympathetic skin response–based studies have shown a variety of autonomic dysfunctions associated with ET, especially in the fields of cardiovascular and urogenital diseases (90–92).

Heart rate variability (HRV) evaluates cardiac autonomic nervous regulation function based on the measurements of beat-to-beat RR variability, including time domains and frequency domains analysis. By measuring the components of HRV analysis in the frequency domain during a 12-h daytime and verifying by DAT-SPECT and cardiac MIBG uptake of 10 ET patients, 10 PD patients, and 10 age-sex-matched controls, investigators found that low-frequency components of HRV analysis helped differentiate between ET and PD (93). In another case-control study enrolling 23 ET patients, 27 TDPD patients, and 23 healthy controls, researchers found that HRV was significantly lower in the TDPD group, and the low-frequency component was the best diagnostic marker (AUC = 0.87) for differentiating ET and PD (94). Another study based on sympathoneural imaging in four family members indicated that overexpression of normal α-synuclein and cardiac sympathetic denervation had an impact on parkinsonism (95).

Heart rate variability analysis as a non-invasive tool and a reflection of autonomic nervous function plays an important role in differentiating ET and TDPD at the early stage of disease (94). However, the results of HRV can be affected by many factors, including age, gender, cardiovascular risk factors, and medications. Even deep brain stimulation can produce certain effect on cardiac electrophysiological activity (96, 97). The analysis of HRV can reflect autonomic nerve regulation of the heart and provide information about sympathetic nerve function status.

A recent clinical study with PD showed that failure to increase total peripheral resistance with cardiac denervation under orthostatic stress was associated with systolic blood pressure reduction leading to orthostatic hypotension (98). Another study that enrolled 75 elderly patients with ET and 25 age-matched controls found no difference between the two groups in orthostatic vital signs, ambulatory 24-h blood pressure monitoring and 24-h Holter monitoring values (99). Therefore, more studies are needed to explore the role of HRV in the development of ET-PD.

## Genetics

Elucidating the genetic background of ET and PD is crucial for understanding the pathogenesis and improving diagnostic and therapeutic strategies. Research has shown that the risk of ET is significantly increased in PD relatives (100, 101); similarly, studies have found that the risk of PD is increased in first-degree relatives of ET patients (102), which indicate that although the two diseases are mostly distinct in their etiology and symptoms, there may be potential genetic pleiotropy between them.

By comparing and analyzing the clinical characteristics of 25 patients with ET-PD and a control group, Ryu et al. (69) found that ET-PD patients had an obvious family history of tremor of first-degree relatives. Abundant genetic studies have revealed that the family history of tremor may be a prodromal marker for PD in ET patients (103).

While a number of studies have failed to identify the causative genes (104–111), several risk genes appear to have an overlapping role in ET and PD (112). For instance, association studies have found that leucine-rich repeat and immunoglobulin containing 1 gene is involved in the pathogenesis of ET and PD (113–118) and serves as a potential therapeutic target in these two disorders. HTRA2, which encodes a serine protease, plays a role in the pathogenesis of genetic type ET (119, 120), and its homozygous allele is involved in the pathogenesis of PD (121), providing genetic evidence of a link between the two disorders, although an Asian study did not find a role for this gene in ET-PD (122, 123). Moreover, using polymerase chain reaction, research has shown that the intermediate copy number of C9ORF72 repeats increases the risk of PD and ET-PD (124). However, a recent study focusing on the genotype excluded 56 samples from analysis as they had genotyping call rates <0.90. There were no variants significantly deviated from Hardy–Weinberg equilibrium (all had *p* > 0.01) (125). Generally, the role of genetic risk factors in neurodegenerative diseases needs to be further explored in larger studies, including genes that can serve as prodromal markers for the development of PD in ET.

## Other Prodromal Markers

Electrophysiological parameters of tremors have the potential to become prodromal markers as technology becomes more clinic-friendly. A study found that the concordance rate between clinical and electrophysiological methods in diagnosing of ET was 94.4% (51 of 54) (126), indicating that electrophysiological methods may be of value in quantifying preclinical patients. One study utilizing an electrophysiological approach to access children and adolescents found an interesting phenomenon: the mean tremor frequency with arms extended was different between children (5.3 Hz) and adolescents (9.0 Hz) (127). This finding suggests that the pathogenesis may be different between children and adolescents. Previous studies have shown an increased R2 recovery of the blink reflex in PD and increased R2 recovery component of the blink reflex (R2-BRrc) in ET associated with resting tremor while normal in ET patients (128). A 2015 study revealed that the probability of the auditory startle reaction was significantly lower in ET-PD patients, whereas it was similar in both healthy subjects and ET or PD patients (*P* < 0.001) (129). Another study that enrolled 19 ET-PD patients and 85 controls (i.e., 48 ET patients and 37 PD patients) found that electrophysiological parameters (i.e., a synchronous resting tremor pattern and the abnormal blink-recovery cycle) were the most accurate biomarkers in distinguishing ET-PD patients from ET or PD patients (130), which calls for longitudinal studies to evaluate whether it can be used as a prodromal marker for the development of PD in ET. Additionally, Crowell et al. (131) utilized an electrocorticography study to address movement disorders such as PD, primary dystonia, and ET with abnormalities in synchronized oscillatory activity. With the development of artificial intelligence and big data, smart phones and sports bracelets can sense and record the characteristics of various parameters of tremor and then enter them into big data analyses (132, 133). Electrophysiological approaches may be new and effective prodromal markers for predicting the development of PD in ET patients.

Studies of behavioral factors in ET and PD are indispensable. For instance, alcohol consumption and dairy intake may be risk factors for PD (134). A study using a population-based, case-control design analyzed ever smokers and never smokers and revealed that ever smokers had less than half the risk of ET (odds ratio = 0.58, 95% confidence interval = 0.40–0.84, *p* = 0.004). The amount of smoking played a subtle role in the development (135). Another study that examined body mass index and waist circumference of PD patients found a possible interaction between anthropometry, sex, and smoking and PD risk (136). Similarly, new prodromal markers are expected to be found when large-scale statistical analyses of patient lifestyle factors are completed.

## Conclusion

**Figure 1:**
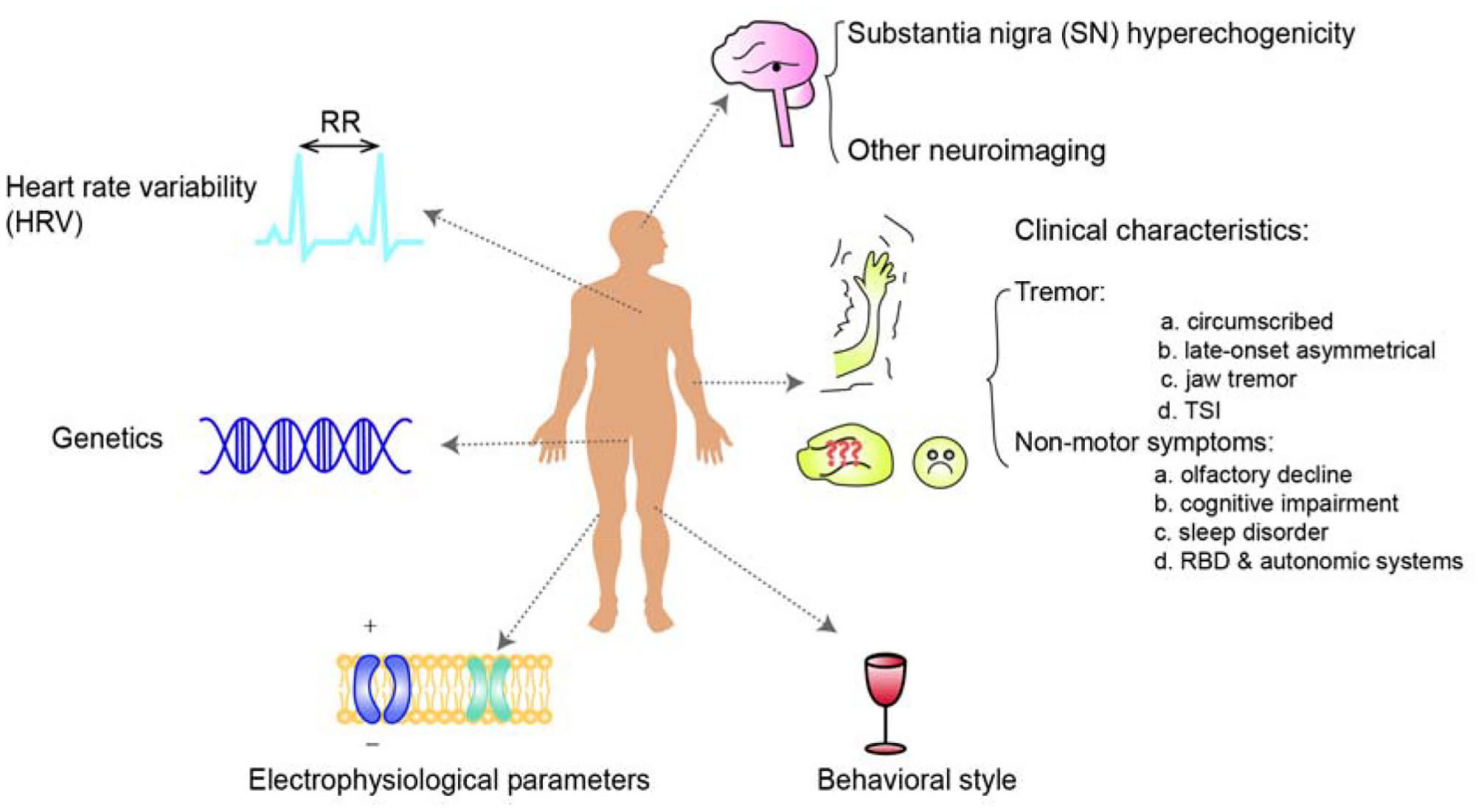
An overview of the risk markers of Parkinson's disease in patients with essential tremor. Essential tremor patients have an increased risk of the development of PD, especially those with SN hyperechogenicity in TCS. Similarly, ET patients with specific tremor or non-motor symptoms, additional HRV, genetic risk, or the other risk markers such as Tremor Stability Index are undergoing a higher risk of the development of PD afterward.

Identification of prodromal markers for the development of PD from ET represents one of the most urgent unmet needs in neurology. This relationship is garnering an increasing amount of interest among researchers. Evidence based on clinical characteristics, epidemiology, neuroimaging, genetics, pathology, and many other aspects has demonstrated numerous associations between these two conditions. Essential tremor patients have an increased risk of developing PD, especially those with SN hyperechogenicity in TCS which is directly demonstrated by a longitudinal study or a prospective cohort study among ET patients. Similarly, ET patients with specific tremor characteristics or non-motor symptoms, HRV, and certain genetic variations are likely to have a higher risk of developing of PD afterward (Figure 1) with the evidence derived from retrospective study, cross-sectional study or prospective study focusing on the characteristics of ET, PD, ET-PD or the differential diagnosis (Table 4). However, given the lack of research directly evaluating PD prodromal markers in ET, we are far from understanding the relationship, and large longitudinal studies of clinical characteristics, genetics, and pathology are needed.

**Table 4:** Brief summary of study design with regard to different prodromal markers.

	**Prodromal markers**	**References**	**Place**	**Study design**	**Number of subjects**	**Main results**
Follow-up ET patients (direct evidence)	SN hyperechogenicity	Sprenger et al. (27)	Austria	Prospective follow-up cohort study Follow-up: mean 6.16 ± 2.05 years	70 ET patients	The relative risk for developing PD in patients with ET who had hyperechogenicity at baseline versus those without this hyperechogenicity was 7.00 (95 CI, 1.62–30.34; sensitivity, 77.8%; specificity, 75.6%)
		Cardaioli et al. (28)	Italy	Longitudinal study Follow-up: 3-year	79 with PD, 59 with ET and 50 matched controls	The maximum size of the SN hyperechogenicity was as follows: 5.62 ± 5.40 mm^2^ in the control group, 19.02 ± 14.27 mm^2^ in patients with PD, 9.15 ± 11.26 mm^2^ in patients with ET-, 20.05 ± 13.78 mm^2^ in patients with ET+ and 20.13 ± 13.51 mm^2^ in patients with ET-PD. ET-PD maximum values were significantly different from controls. Maximum values in patients with ET+ were different from both controls and patients with ET
Explore the characteristics of ET, PD, or ET-PD patients	SN hyperechogenicity	Budisic et al. (26)	Croatia	Case-control study	80 PD patients, 30 ET patients, and 80 matched controls	Bilateral SN hyperechogenicity over the margin of 0.20 cm (2) was found in 91% of PD patients, 10% of healthy subjects, and in 13% patients with ET
Patients or make differential diagnosis (partial lateral evidence)	Circumscribed resting tremor	Minen and Louis (44)	USA	Retrospective study	53 ET-PD, 53 PD and 150 ET patients	The initial cardinal sign of PD was rest tremor in 100% of patients. In ET-PD, the side of greatest initial ET severity usually matched that of greatest PD severity (*P* < 0.05)
	Late onset asymmetrical postural tremor	Chaudhuri et al. (45)	UK	Longitudinal study	13 ET-PD patients	After a variable and long latent period all patients developed additional signs suggesting a clinical diagnosis of PD although picking up an initial label of ET
	TSI	di Biase et al. (48)	Italy	Cohort study	16 TDPD and 20 ET patients	TSI with a cut-off of 1.05 gave good classification performance for PD tremor and ET, in both test and validation datasets
	Olfactory decline	Louis et al. (50)	USA	Case-control study	83 ET patients and 69 controls	In 83 ET cases, higher log blood harmane concentration was correlated with lower UPSIT score (rho = −0.46, *p* < 0.001)
	Cognitive decline	Louis et al. (53)	USA	Clinical-epidemiological study	30 ET-PD and 53 age-matched PD patients	The MMSE score was lower in ET-PD than PD [26.5 ± 3.1 (median 28.0) vs. 28.4 ± 2.2 (median 29.0), *p* = 0.001]. The TICS score was lower in ET-PD than PD [31.7 ± 3.9 (32.0) vs. 35.0 ± 2.0 (35.0), *p* < 0.001]
	Sleep disorder, especially RBD	Lacerte et al. (87)	Canada	Cross-sectional study	50 ET patients	Using a screening questionnaire for RBD, 43.5% of ET patients are possibly suffering from RBD, whereas in the general population prevalence is estimated to be 0.5%
	HRV	Yoon et al. (94)	South Korea	Case-control study	23 with ET, 27 with TDPD and 23 healthy controls	In the TDPD group, SDNN, LF, HF, and TP were significantly lower than those in the ET group. In a receiver operating characteristic AUC analysis, LF was the best potential diagnostic marker (AUC = 0.87)
	Others	Yavuz et al. (129)	Turkey	Case-control study	15 ET, 7 ET with resting tremor, 25 ET-PD, 10 PD and 12 healthy subjects	Probability of ASR was significantly lower in ET-PD group whereas it was similar to healthy subjects in ET and PD (*P* < 0.001). LLR II was more common in ET, PD and ET-PD groups. LLR III was far more common in the PD group (*n* = 3, 13.6% in ET; *n* = 4, 16.0% in ET-PD and *n* = 7, 46.7% in PD; *p* = 0.037)

*ET, essential tremor; SN, Substantia nigra; PD, Parkinson's disease; ET-PD, new-onset of PD in patients previously diagnosed with ET; ET+, ET developed new-onset parkinsonian features, without fulfilling criteria for PD diagnosis; ET-, ET patients did not develop parkinsonian features; TSI, Tremor stability index; TDPD, tremor-dominant PD; UPSIT, the University of Pennsylvania Smell Identification Test; MMSE, Mini-Mental State Examination, TICS, Telephone Interview for Cognitive Status; RBD, REM sleep behavior disorder; HRV, Heart rate variability; SDMN, standard deviation of the normal-to-normal RR interval; LF, low-frequency; HF, high-frequency; TP, total spectral power; AUC, area under the curve; ASR, auditory startle reaction; LLR, long latency reflex*.

## Author Contributions

Y-CW provided fund support, revised the manuscript, and designed the project ideas. X-XW, YF, and XL searched for literature and wrote the manuscript. X-YZ, DT, and WO revised the paper. All authors contributed to the article and approved the submitted version.

## Conflict of Interest

The authors declare that the research was conducted in the absence of any commercial or financial relationships that could be construed as a potential conflict of interest.

## Abbreviations

ET: essential tremor
PD: Parkinson's disease
RBD: rapid eye movement sleep behavior disorder
SN: substantia nigra
TCS: Transcranial sonography
TSI: Tremor Stability Index
RBDSQ: RBD screening questionnaire
pRBD: probable RBD
SS-16: 16-item Sniffin' Sticks test
HRV: Heart rate variability
TDPD: tremor-dominant PD.
